# Study of the oxidative stability via Oxitest and Rancimat of phenolic-rich olive oils obtained by a sequential process of dehydration, expeller and supercritical CO_2_ extractions

**DOI:** 10.3389/fnut.2024.1494091

**Published:** 2024-11-26

**Authors:** Assamae Chabni, Celia Bañares, Carlos F. Torres

**Affiliations:** ^1^Department of Production and Characterization of Novel Foods, Institute of Food Science Research (CIAL, CSIC-UAM), Autonomous University of Madrid, Madrid, Spain; ^2^Department of Bioactivity and Food Analysis Institute of Food Science Research (CIAL, CSIC-UAM), Autonomous University of Madrid, Madrid, Spain

**Keywords:** accelerated oxidation methods, expeller, kinetic analysis, olive oil, shelf life, supercritical CO_2_ extraction

## Abstract

The oxidative stability of olive oils extracted by different methods, i.e. conventional 2-phase extraction (cOO), and sequential extraction by expeller press (eOO) and supercritical CO_2_ (SCOO), was determined by using two accelerated oxidation methods, Oxitest and Rancimat, in the temperature range 90–160°C. The kinetic analyses carried out provided Arrhenius activation energies, enthalpies, entropies and Gibb’s free energies of activation, temperature coefficients, Q_10_ factors, and the oxidative stability indexes at 20°C (OSI_20_) for the different oils. A good correlation between the two techniques was obtained (r^2^ = 0.996). Oxitest showed, however, shorter induction times and less sample quantity (1 g vs. 3 g in Rancimat) requirements, suggesting that it could be a good and faster alternative to Rancimat for the evaluation of the oil oxidative stability. cOO showed OSI_20_ values of 38.5 and 42.5 months, by the Rancimat and Oxitest methods, respectively. Furthermore, eOO and SCOO showed OSI_20_ values of 43.3 and 138.6 months by Rancimat and 67 and 142 months by the Oxitest method, respectively. The strong correlation found between the phenolic content of the oils and their OSI_20_ values confirms that a higher oil phenolic content would improve the oxidative stability of the oils.

## 1 Introduction

Virgin olive oil (VOO) is one of the few oils that are consumed without refining. It is also one of the most oxidation-resistant vegetable oils due to its fatty acid profile, composed mainly by monounsaturated fatty acids (MUFAs), which make them highly stable, and, additionally, a high content of minor antioxidant compounds (polyphenols and tocopherols) (1). However, once oxidation begins, undesirable changes develop in the oil that affect its quality and produce compounds that are potentially harmful to health (2). This is known as oxidative rancidity, and it is the most important quality factor to be considered, since it has a negative impact on the organoleptic and nutritional properties of the oil, causing unacceptable off-flavors, changes in color and texture, and a decrease in the content of vitamins, minority compounds and healthy free fatty acids (3).

**Figure 1:**
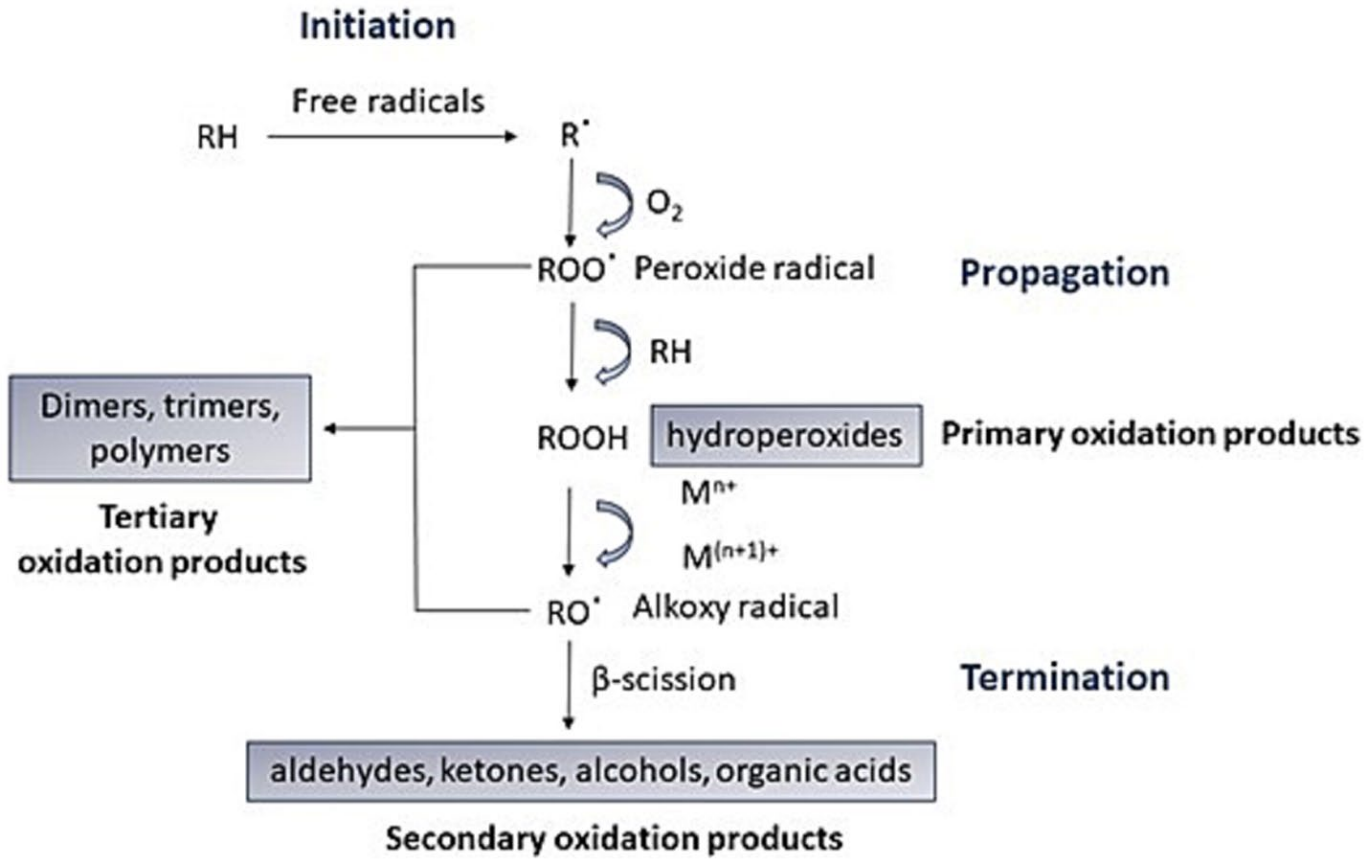
Auto-oxidation mechanism of lipids with initiation, propagation, and termination steps. Unsaturated fatty acid (RH), radical (R·), peroxide radical (ROO·), hydroperoxides (ROOH), Metal (Mn+), alkoxyl radical (RO·) (1).

Olive oil (OO) oxidation begins when it comes into contact with oxygen (O_2_) during production and storage (1), involving three stages: initiation, propagation, and termination (Figure 1). In the initiation step, a free radical causes polyunsaturated fatty acid (PUFAs), like linoleic acid, to lose a hydrogen atom, forming an alkyl radical (R·). During propagation, this radical reacts with O_2_ to form a peroxide radical (ROO·), which then reacts with another PUFA to create a hydroperoxide (ROOH), which is a primary oxidation product. The decomposition of ROOH is influenced by the number of C=C double bonds in the fatty acid, with linoleate ROOH decomposing faster than oleate ROOH. ROOH is unstable and, in the presence of metals, forms alkoxyl radicals (RO·), leading to secondary oxidation products like aldehydes and ketones. The chain reaction ends when radicals combine, forming non-volatile compounds like dimers and polymers (tertiary oxidation products). Oxidation can also occur via photo-oxidation and thermo-oxidation in the presence of light or heat, respectively (4).

Initially, oxidation of VOO is slow because the antioxidant compounds in the oil itself deactivate free radicals production, thus preventing autoxidation (1). These antioxidant compounds, mainly polyphenols, are of vital importance as they play an important role in the nutritional value of VOO and contribute greatly to the shelf life of the oil by improving its oxidative stability (5). However, the content of phenolic compounds (PC) in the oil depends strongly on the extraction process employed (6, 7, 61). Miho et al. (8) have found that malaxation process negatively affects the content of PCs in OO because they are hydrolyzed, resulting in less stable derivatives. Meanwhile, Marx et al. (9) and Novoselić et al. (7) have shown that the use of water during the extraction of OO reduces the content of PC in the oil, as these are lost in the oil mill water due to their amphiphilic character.

In a previous study on the influence of water on the extraction of OO (10), it was found that oils extracted from dehydrated olives by expeller press show a higher content of total phenolic compounds (TPC) compared to those extracted in the presence of water, and their oxidative stability at 120°C increases proportionally. Therefore, one way to significantly increase the PC content in OO is through a two-step sequential extraction process, combining expeller press and supercritical fluid technology after removing the water from the olives, to obtain two different OOs, one by expeller extraction and the other by supercritical CO_2_ extraction (11, 12). The absence of water avoids the hydrolysis of PCs, such as oleuropein and lignostrides, as well as their loss in the oil mill water or in the pomace. Therefore, they are found in higher concentration in the oil, preventing future peroxidation propagation reactions or eliminating free radicals.

To determine the degree of oxidation of OO, legislation (13) establishes the peroxide value to determine the oxidative state of the oil after extraction. The peroxide value indicates the content of hydroperoxides, providing an estimate of the overall oxidative state of the oil, particularly in the primary oxidation phase. In addition, legislation also establishes the ultraviolet specific extinction coefficients (K_232_ and K_270_) obtained by spectrophotometric measurements to determine the conjugated dienes and trienes formed during the refining process (14). However, the extinction coefficients K_232_ and K_270_ must be taken with caution, since dienes and trienes are not the only absorbers at these wavelengths. PCs contribute strongly to absorption between 220 and 380 nm. In this wavelength range, the UV spectra of a diluted OO in hexane and the methanol–water extracted PCs from OO are comparable (15, 16). In the present study, the focus is also placed on the p-anisidine value (AnV), as it provides information about the oxidation status of the oil based on the concentration of aldehydes and ketones, which are secondary oxidation products (17).

The antioxidant content of OO decreases during storage. Therefore, there are methods and oxidation detection techniques to determine OO resistance to oxidation caused by temperature, light, and oxygen, that are utilized to determine OO shelf life (3). Some of these techniques are Schaal, Rancimat and Oxitest, among others (18). Due to the time-consuming Schaal oven test where oils are stored at 60°C for weeks, accelerated methods such as Rancimat and Oxitest are better accepted (2). The Rancimat test forces the oxidation of the fatty acids in the oil by means of a continuous air stream, resulting in the formation of peroxides as primary oxidation products. After a period of time, due to the action of temperature and air flow, secondary oxidation products, such as acetic acid and formic acid, which are low molecular weight volatile organic acids, are also produced. These are diluted in water increasing its conductivity. When the system reaches the maximum change in conductivity, the induction time or oxidative stability index (OSI) (in hours) is determined (18). The Oxistest (Oxidation Test Reactor) monitors the oxygen uptake of the reactive components present in the lipid matrix (solid or liquid) to evaluate the oxidative stability under accelerated oxidation conditions at temperatures up to 120°C and oxygen pressure of 8 bars (19).

The present work compares the oxidative stability of a commercial VOO and different OOs obtained by conventional two-phase extraction process, by expeller press from dehydrated olives (10), and by supercritical CO_2_ extraction from the press cake, obtained via Rancimat and Oxitest methods. OO extraction technologies play a crucial role in the final quality of the product, particularly in the preservation of phenolic compounds, which are known for their antioxidant properties. The conventional two-stage process is one of the most widely used in the olive oil industry, as it combines high efficiency with the preservation of sensory and nutritional characteristics of the oil. However, expeller press extraction from dehydrated olives avoids water utilization, which can influence the concentration of natural antioxidants. On the other hand, supercritical CO_2_ extraction, a more advanced and environmentally friendly technology, allows obtaining oils with a different bioactive compound profile by avoiding exposure to high temperatures and chemical solvents. These technological differences have direct implications on the oxidative stability of the oils and, eventually, on their quality and shelf life. This study not only evaluates these methodologies, but also establishes the relationship between oxidative stability and the content of total phenolic compounds in the oils obtained. Arrhenius activation energies and pre-exponential factors, activation thermodynamic magnitudes (enthalpy, entropy and Gibbs free energy), OSI values at 20°C (OSI_20_), temperature coefficients, and Q_10_ values, have been obtained from the kinetic studies.

## 2 Materials and methods

### 2.1 Materials

Olives from Cornicabra variety produced at the end of the 2020/2021 crop season provided by Arganda oil cooperative, Arganda del Rey, province of Madrid (Spain). Commercial virgin olive oil (VOO) was purchased from a local market and stores at room temperature under dark conditions until the analyses. For the extraction by supercritical fluids, carbon dioxide (CO₂) with a purity of 99.9% was acquired from Carburos Metalicos (Barcelona, Spain). Solvents used were hexane (HEX), methyl-tertbutyl ether (MTBE), isopropyl alcohol (ISOP), methanol (MeOH), and chloroform (CLF), which were supplied by Macron (Avantor Performance Material, Center Valley, PA, USA). Reagents used for determinations as phenolphthalein, Folin–Ciocalteu reagent and sodium carbonate (Panreac, Barcelona, Spain), boron trifluoride methanol complex solution (BF3) 14% in methanol from Sigma-Aldrich (St. Louis, USA), and hydroxytyrosol (HT) standard ≥98% were supplied by Seprox Biotech (Madrid, Spain). Peroxide and p-anisidine reagents were supplied by CDR FoodLabFat (Florence, Italy).

### 2.2 Olive oil extraction, initial status, fatty acid profile and total phenolic content

The extraction of olive oils was carried out following the procedures of a previous study, in which the oils were also fully characterized. Three different types of oils were extracted, a control OO (cOO) obtained by the conventional two-phase extraction system from raw olives, an expeller OO (eOO) and a supercritical CO_2_ OO (SCOO) (11), both oils obtained by a sequential extraction process combining expeller press and supercritical fluids from dehydrated olives. The extractions were carried out in duplicate.

The initial quality status, fatty acid profile (FA) and total phenolic content (TPC) of the extracted oils and commercial VOO were determined. Briefly, the quality indexes (acid value, peroxide value, absorption coefficients K_232_ and K_270_, and adulteration ratio ΔK) were determined according to the Commission Regulation (EEC) No 2022/2105 (U, 2022) on the properties of olive oil and olive residue, thus confirming that the latter (VOO) is of the virgin category (Table 1). The peroxide (PV) stated as milliequivalents of active oxygen per kilogram of oil (meq O2 kg^−1^) was measured as primary oxidation indicator, and the p-anisidine value (AnV), together with extinction coefficient (K_270_), were measured as secondary oxidation indicators. These indicators (PV and AnV) were measured based on colorimetric reactions using a CDR FoodLabFat (Florence, Italy) following the manufacturer’s instructions, according to official Methods of AOCS, methods Cd 8–53 and Cd 18–90 (20), through the measurement of the absorbance at 505 nm and 366 nm for PV and AnV, respectively. In addition, the total oxidative deterioration was evaluated by calculating the TOTOX value according to:

(1) TOTOX Value=AnV+2PV

**Table 1:** Quality indexes: acidity value (AV), peroxide value (PV), extinction coefficients (K_232_ and K_270_), p-anisidine value (AnV), and TOTOX value for cOO, eOO, SCOO and VOO.

	VOO	cOO	eOO	SCOO	EVOO*
AV (% oleic acid)	0.95 ± 0.05^a^	0.68 ± 0.11^b^	0.57 ± 0.07^b^	0.75 ± 0.06^b^	≤ 0.8
PV (meq O_2_/kg oil)	11.1 ± 1.3^a^	7.93 ± 0.61^b^	5.51 ± 0.59^c^	5.86 ± 0.12^c^	≤ 20
K_232_	2.02 ± 0.01^a^	1.86 ± 0.01^b^	1.78 ± 0.0^c^	1.75 ± 0.05^b^	≤ 2.50
K_270_	0.25 ± 0.01^a^	0.19 ± 0.01^b^	0.18 ± 0.0^b^	0.18 ± 0.01^b^	≤ 0.22
ΔK	0.01 ± 0.0^a^	0.01 ± 0.0^a^	−0.006 ± 0.0^b^	−0.01 ± 0.0^b^	≤ 0.01
AnV	2.80 ± 0.44^a^	2.50 ± 0.62^a^	2.93 ± 0.52^a^	4.30 ± 0.2^b^	–
TOTOX	25 ± 3.04^a^	17.6 ± 0.37^b^	13.9 ± 0.73^c^	16.2 ± 0.08^b^	–

Significant differences are indicated in the same row with different letters (*p* < 0.05).*EVOO: extra virgin olive oil quality criteria, value limits set by European Union.

The fatty acid methyl esters (FAME) were obtained according to the AOAC Official Method 996.01 (21) and analyzed by gas chromatography (11). The TPC of the obtained oil was determined by the Folin–Ciocalteu spectrophotometric method at 760 nm, using hydroxytyrosol (HT) within the range of 0–1 mg L^−1^ calibration curve (22). Total phenolic compounds were given as mg of HT per kg of oil (mg HT/kg). These determinations were carried out in duplicate for each sample.

### 2.3 Rancimat and Oxitest methods

Before carrying out the oxidative stability kinetic study of the extracted oils, the correlation between the Rancimat and Oxitest methods was studied using a commercial VOO. In addition, an optimization step was implemented to find the appropriate sample quantity for Oxitest analyses. For this purpose, the VOO was oxidized at 90°C, at an oxygen pressure of 6 bar, and sample amounts of 1, 3 and 10 g. For the correlation between the two methods, the same temperature (T) range (90–120°C) was used.

Oxidative stability of cOO, eOO and SCOO was determined by using a Rancimat apparatus (743 Rancimat, Metrohm, Hesirau, Switzerland). Oil samples of 3 ± 0.01 g under a constant air flow (15 L h − 1) were used in the T range 90–140°C for cOO, 100–140°C for eOO, and 110–160°C for SCOO, with variations of 10°C. The induction period or oil stability index (OSI) was automatically registered as the proper endpoint, which was taken as the intersection point of the extrapolated curves (or plotted curves break point) (19).

The Oxitest Cd 12c-16 (Firestone, 2009) is an oxidation stability reactor (VELP Scientifica, Usmate Velate (MB), Italy) that allows rapid measurements of the stability of foods against lipid oxidation by subjecting the sample to high T and pure oxygen overpressure. In the present investigation, 1 g of the different olive oils was analyzed at different working Ts (90, 100, 110 and 120°C) and at a pressure of 6 bar. OSI values were obtained from the pressure–time curves measured, as the time required to reach a 10% drop in oxygen pressure inside the oxidation chamber. The Oxitest software calculates a linear regression equation on a semi-logarithmic scale (logarithm of the OSI-T curve) to predict the estimated OSI and thus the shelf life of the products at the desired storage T (20°C) (OSI_20_). All measurements were performed in duplicate.

### 2.4 Antioxidant activity index

The antioxidant activity of the different oils studied in this work was obtained according to the antioxidant activity index (AAI) (23) defined as:

(2) $$AAI=\frac{{OSI}_{s}}{{OSI}_{c}}$$

where OSI_S_ and OSI_C_ correspond to the OSI values of the obtained olive oils and that of the commercial VOO, respectively. The protective effect of the antioxidant corresponds to AAI > 1, whereas AAI = 1 indicates non protective effect, and the pro-oxidant effect is related to AAI < 1.

### 2.5 Kinetic data analysis

The well-known Arrhenius equation (24) was used to determine activation energies (Ea, kJ/mol) and pre-exponential factors (A, h–1), from the slope and intercept according to:

(3) $$\ln\;k=\ln\;A-\frac{E_{a}}{RT}$$

where k represents the reaction rate constant, which in the present case corresponds to the reciprocal OSI (h–1), i.e., k = 1/OSI, R is the molar gas constant (8.314471 Jmol^−1^ K^−1^), and T is the temperature in K.

The activation thermodynamic magnitudes are obtained from the thermodynamic formulation of the transition state theory (24) using the equation:

(4) $$\ln\left(\frac{1}{OSI}\frac{1}{T}\right)=\ln\left(\frac{k_{B}}{h}\right)+\frac{\Delta S^{‡}}{R}-\frac{\Delta H^{‡}}{R}\frac{1}{T}$$

where *Δ*S^‡^ and ΔH^‡^ are the activation entropy and enthalpy, respectively, and they are obtained from the slope and the intercept of the linear regression ln(1/OSI·1/T) vs. 1/T. In Equation 4, h is the Planck constant (6.62608 × 10^−34^ J K^−1^), kB is the Boltzmann constant (1.38065 × 10^−23^ J K^−1^) and R is the molar gas constant. The Gibb’s free energy of activation (ΔG^‡^) was obtained from the fundamental thermodynamics’ equation:

(5) $$\Delta G^{‡}=\Delta H^{‡}-T\Delta S^{‡}$$

Temperature coefficients (tcoeff, oC) are obtained from the linear regression log (OSI) vs. temperature (t, oC), following the equation:

(6) logOSI=B+At

where the slope (A) corresponds to tcoeff and B is the intercept. Finally, the temperature acceleration factor Q10, defined as the increase in oxidation rate by a 10°C increase in temperature, was calculated as (OSIt)/(OSIt +10°C). If the reaction rate doubles with the change of 10°C with temperature, Q_10_ = 2.

### 2.6 Statistics

Data analysis was performed using Excel (Microsoft Office 365) and all statistical evaluations were performed using Origin (version 9.0 for Windows; OriginLab Corporation, Northampton, MA, United States). Experiments were carried out in duplicate, and the data were expressed as mean ± standard deviation. The statistical significance of the differences between the groups was measured using one-way analysis of variance (ANOVA) and *post hoc* Tukey HSD test. Statistical significance was defined at the level of *p* < 0.05.

## 3 Results and discussion

### 3.1 Quality indexes and oxidative status of the oils

The quality indexes and oxidative status of the oils are shown in Table 1. Before the oxidation experiments, the oils had quality indexes, acidity value (AV), peroxide value (PV), absorption coefficients K_232_ and K_270_, and adulteration coefficient (ΔK), which were within the limits established by current legislation (U, 2022) to commercially classify them as virgin OO in the case of the commercial oil (VOO) and as extra virgin OO in the case of those extracted for the study (cOO, eOO, and SCOO). The results showed no significant differences between the oils extracted by the different methods, conventional 2-phase extraction (cOO), expeller press (eOO) and supercritical CO2 extraction (SCOO). The oils also showed good oxidation status, since the overall oxidation and oil quality indicator (TOTOX), as a combination of PV, which measures the amount of primary oxidation products (hydroperoxides), and p-anisidine value (AnV), which measures the secondary products (mainly, alkenals and alkadienals), were lower than the reference value of 26 suitable for human consumption (25).

### 3.2 Oxitest method optimization

The sample quantity to be analyzed by the Oxitest method has been optimized to provide higher versatility and applicability for lipids of high added value. The OSI values obtained for VOO oxidized at 90°C at an oxygen pressure of 6 bar and for sample quantities of 1, 3 and 10 g are shown in Table 2. The OSI values for the different sample weights are in the range 24.5–25.3 h. Therefore, the amount of oil used in the Oxitest method does not show significant differences (*p* = 0.454).

**Table 2:** Oxidative stability index (OSI) of samples of 1, 3, and 10 g for commercial virgin olive oil (VOO) determined by the Oxitest method with oxygen pressure of 6 bar and temperature of 90°C.

Oil weight	OSI (h)
1 g	25.3 ± 0.7
3 g	25.0 ± 0.3
10 g	24.5 ± 0.1

These results are consistent with those reported by Tsao et al. (26) for linseed oil at 110°C, where no significant differences were found between OSI values obtained by using samples of 3, 5, 7 or 10 g. Comandini et al. (27) also found no significant differences in oxidizing samples of 2, 5 and 10 g for extra VOO, and samples of 3, 5, 7 and 10 g for sunflower oil at 110°C. The optimal amount to carry out the Oxitest experiments has been established then in 1 g of sample, since we are used to work with structured or high value-added lipids, which are usually available in small quantities.

**Figure 2:**
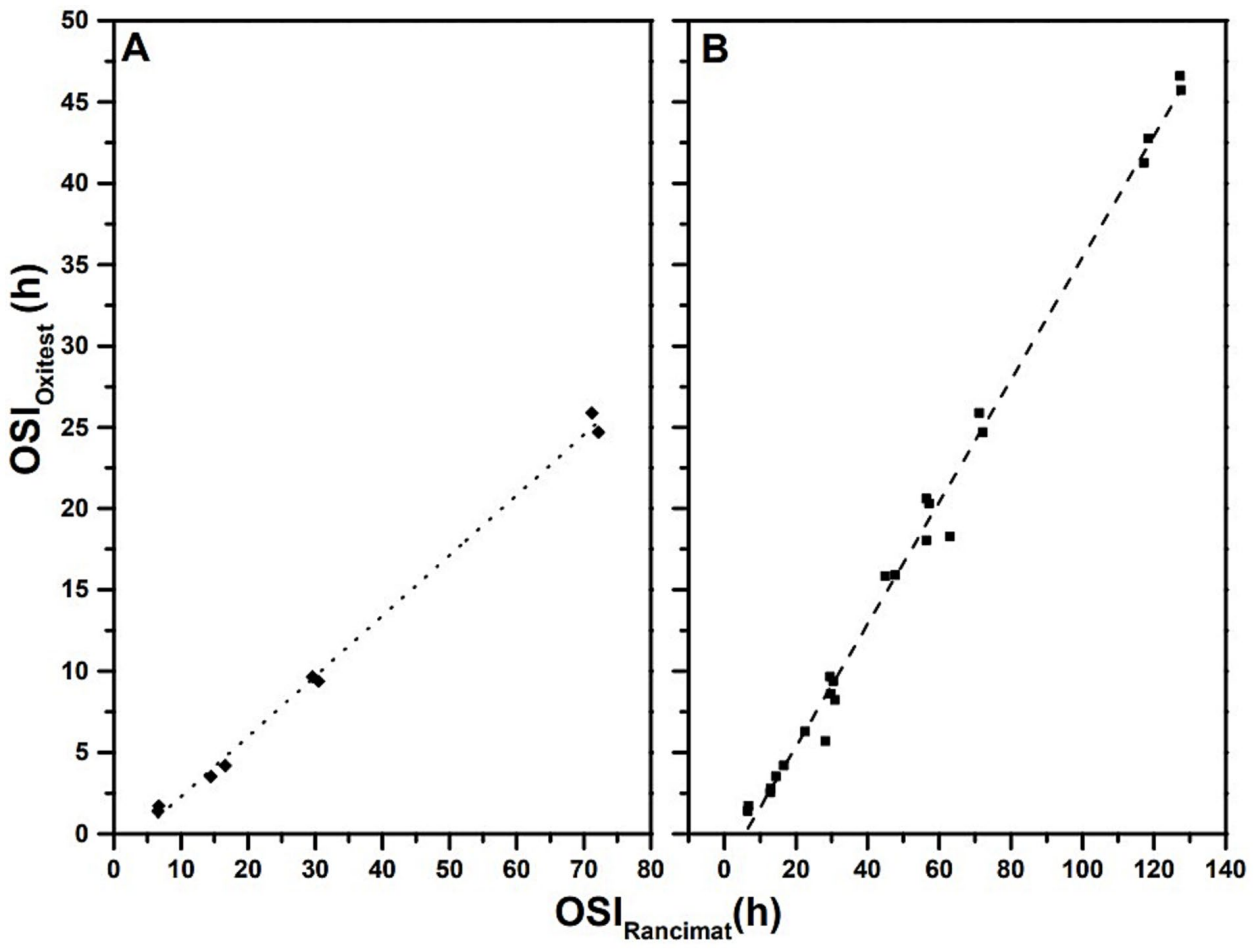
Correlation between the OSI values of VOO (A) and all studied oils (B) obtained by Rancimat and Oxitest methods. A: Y = 0.3707 X – 1.422, r^2^ = 0.996. B: Y = 0.3756 X – 2.238, r^2^ = 0.993.

**Figure 3:**
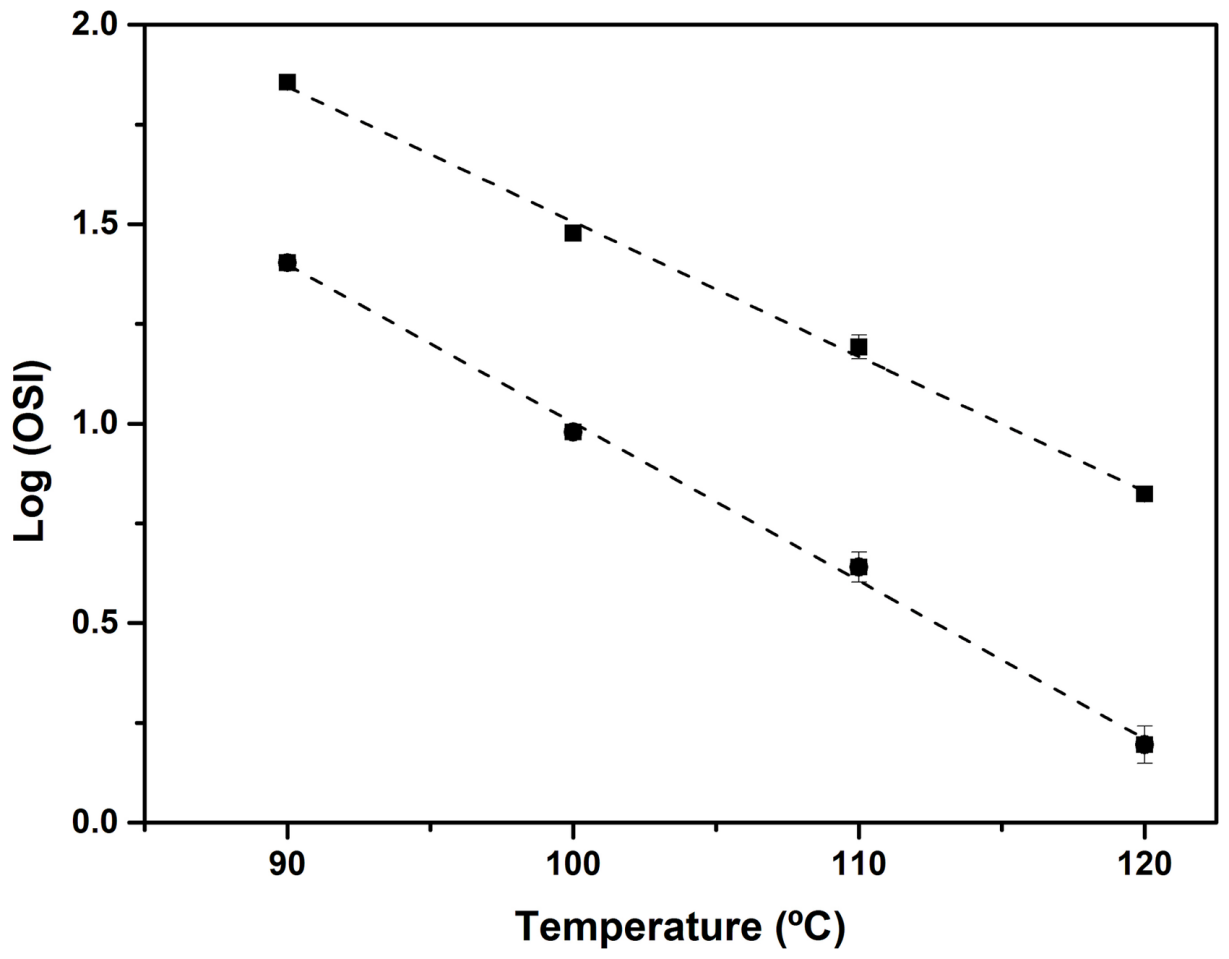
Representation of log (OSI) versus temperature (in °C) for VOO by using Rancimat (squares) and Oxitest (circles) from the data shown in Table 3. Linear regression parameters A (slope) and B (intercept) from Equation 6 are also shown in Table 3.

Figure 2 shows the correlation between the OSI values found for VOO using the two methods, Rancimat and Oxitest. As can be seen, there is a very good correlation (*r* = 0.999, *r*^2^ = 0.996, *p* = 1.5 10^−8^). However, it should be noted that the OSI values obtained by Oxitest are approximately 3.5 times lower than those obtained by the Rancimat method (Figure 3; Table 3). Oxitest then provides similar results to Rancimat, but using a smaller amount of sample and reducing the time for the analysis of the oxidative stability of fat-rich matrices. The better performance of Oxitest may be due to the shorter start-up time and the fact that the measured OSI values correspond to an early stage of lipid oxidation, which is associated to the formation of lipid hydroperoxides. In contrast, Rancimat records the formation of volatile carboxylic acids, which are associated with secondary oxidation reactions (2). This confirms the results obtained by Tinello et al. (19) for frying oils. Moreover, this good correlation is confirmed when using the rest of the oils under study (*r* = 0.997, r^2^ = 0.993, *p* = 0) (see Figure 2), whose shelf life will be discussed later.

**Table 3:** Rancimat and Oxitest oxidative stability indexes (OSI) (in hours) measured in the temperature range 90–120°C (every 10°C) for commercial VOO.

	Rancimat method	Oxitest method
Temperature (°C)	OSI (h)	
90	71.7 ± 0.5	25.3 ± 0.6
100	30.0 ± 0.5	9.5 ± 0.1
110	15.6 ± 1.1	4.4 ± 0.2
120	6.7 ± 0.1	1.6 ± 0.2
log OSI = A*t* + *B* Equation 6		
*A* = *t_coeff_* (^o^C^−1^)	−0.034 ± 0.000	−0.040 ± 0.001
*B*	4.879 ± 0.022	4.972 ± 0.094
*R* ^2^	0.998 ± 0.000	0.996 ± 0.003
*Q*_10_	2.18 ± 0.19	2.54 ± 0.32
OSI_20_ (months)	21.9 ± 1.0	22.2 ± 2.2

The parameters *A* (slope) and *B* (intercept) and the correlation index *R*^2^ obtained from the linear regression of the data to Equation 6 for both methods are indicated along with the temperature coefficient (*t*_coeff_ = A) (in °C^−1^), and Q_10_ and OSI_20_ values. The OSI20 values are the extrapolation of Equation 6 to 20°C.

### 3.3 Fatty acid profile of extracted oils

Analysis of fatty acid (FA) profiles was conducted on oils extracted through conventional 2-phase extraction (cOO), expeller press (eOO), and supercritical CO2 extraction (SCOO). Table 4 summarizes the mean and standard deviation of the FA profiles for the three oils. Notably, no significant differences (*p* > 0.05) were observed in FA composition based on the extraction method used.

**Table 4:** Fatty acid (FA) profile (expressed in g/100 g) for cOO, eOO and SCOO.

g/100 g	cOO	eOO	SCOO	EVOO *
Palmitic acid C16:0	9.15 ± 0.06	9.06 ± 0.00	9.34 ± 0.06	7.00–20.0
Palmitoleic acid C16:1	0.83 ± 0.04	0.80 ± 0.00	0.83 ± 0.00	0.6–3.2
Stearic acid C18:0	3.50 ± 0.02	3.50 ± 0.03	3.53 ± 0.03	0.50–5.00
Oleic acid C18:1 c9	79.96 ± 0.19	80.31 ± 0.04	79.24 ± 0.17	55.0–85.0
Linoleic acid C18:2	4.44 ± 0.02	4.24 ± 0.16	4.48 ± 0.14	2.50–21.0
γ-Linolenic acid C18:3	0.50 ± 0.03	0.49 ± 0.00	0.51 ± 0.01	≤1.00
α-Linolenic acid C18:3	0.93 ± 0.05	0.95 ± 0.03	1.20 ± 0.01	≤1.5
Others	0.70 ± 0.03	0.80 ± 0.04	0.88 ± 0.01	
Saturated FA	12.68 ± 0.05	12.62 ± 0.03	12.92 ± 0.02	
Monounsaturated FA	80.87 ± 0.15	81.18 ± 0.04	80.15 ± 0.17	
Polyunsaturated ω- 6	4.44 ± 0.02	4.24 ± 0.16	4.48 ± 0.14	
Polyunsaturated *ω*- 3	1.42 ± 0.09	1.44 ± 0.03	1.71 ± 0.01	
Ratio O/L	18.00 ± 0.13	18.97 ± 0.72	17.71 ± 0.57	

O/L ratio: oleic/linoleic acid ratio. *EVOO: extra virgin olive oil quality criteria, value limits set by European Union.

Key factors influencing FA composition include latitude, climate, variety, and oil maturity. Cornicabra oils are characterized by their high oleic acid content (76.5–82.5%) and low linoleic acid content (3.1–6.6%) (28), characteristics that are consistent with the results of previous research on the same olive variety (29). The FA composition of cOO, eOO, and SCOO includes between 9.06–9.34% palmitic, 3.5–3.53% stearic, 79.24–80.31% oleic, 4.24–4.48% linoleic, and 1.42–1.71% linolenic acids. Furthermore, it has been observed that olive dehydration does not affect the FA composition of the oils, in agreement with previous studies (10, 30–32). Similarly, oils obtained by CO_2_-SFE exhibit the same FA composition as those extracted by conventional methods, as indicated by other authors (33).

The oleic-to-linoleic (O/L) ratio (see Table 4) is indicative of the oxidation stability of the olive oil (34). The O/L ratios obtained in this work are in good agreement with those reported by Román Falcó et al. (35) for cornicabra variety oils (17.7–18.9). When the O/L ratio is low, i. e. when the oleic acid content is low compared to the linoleic acid content, the stability of the oil and therefore its shelf life could be affected (36), since it has been observed that 24% of the FA composition contributes to oxidative stability measured by Rancimat (37).

### 3.4 Total phenolic content of extracted oils

The phenolic compound (PCs) content of the extracted oils was 118.7 ± 9.1, 416 ± 157, and 1917 ± 334 mg EHT/kg product for cOO, eOO and SCOO, respectively. As discussed in previous studies (10), the extraction method used determines the amount of PCs and justifies the significant difference in the content of PCs in the oil.

When olives are dehydrated and extracted by expeller press, the PCs are distributed between the oil (eOO) and the press cake, and then when this press cake is extracted by SFE-CO_2_, the PCs distribute between the defatted flour and the oil (SCOO). On the other hand, if conventional OO (cOO) extraction is used together with water, the PCs remain mostly in the aqueous residues due to their hydrophilicity (38, 39).

Virgin olive oil is the only oil that contains significant amounts of natural antioxidants (phenols and tocopherols), which, in addition to contributing to the characteristic fruity and bitter taste of these oils, are largely responsible for the oxidative stability of the OOs (40). Moreover, the content of PCs and, consequently, the oxidative stability of the OO also depend on the variety. All the oils analyzed contain considerable amounts of PCs. The PCs achieved for cOO are similar to those obtained by other authors for oils of the same variety (41). eOO and SCOO have a high content of PCs, determining the oxidative stability, as they act as primary antioxidants to inhibit oxidation in VOO (1).

### 3.5 Oxidative stability, estimated shelf-life and antioxidant capacity of the extracted oils

**Figure 4:**
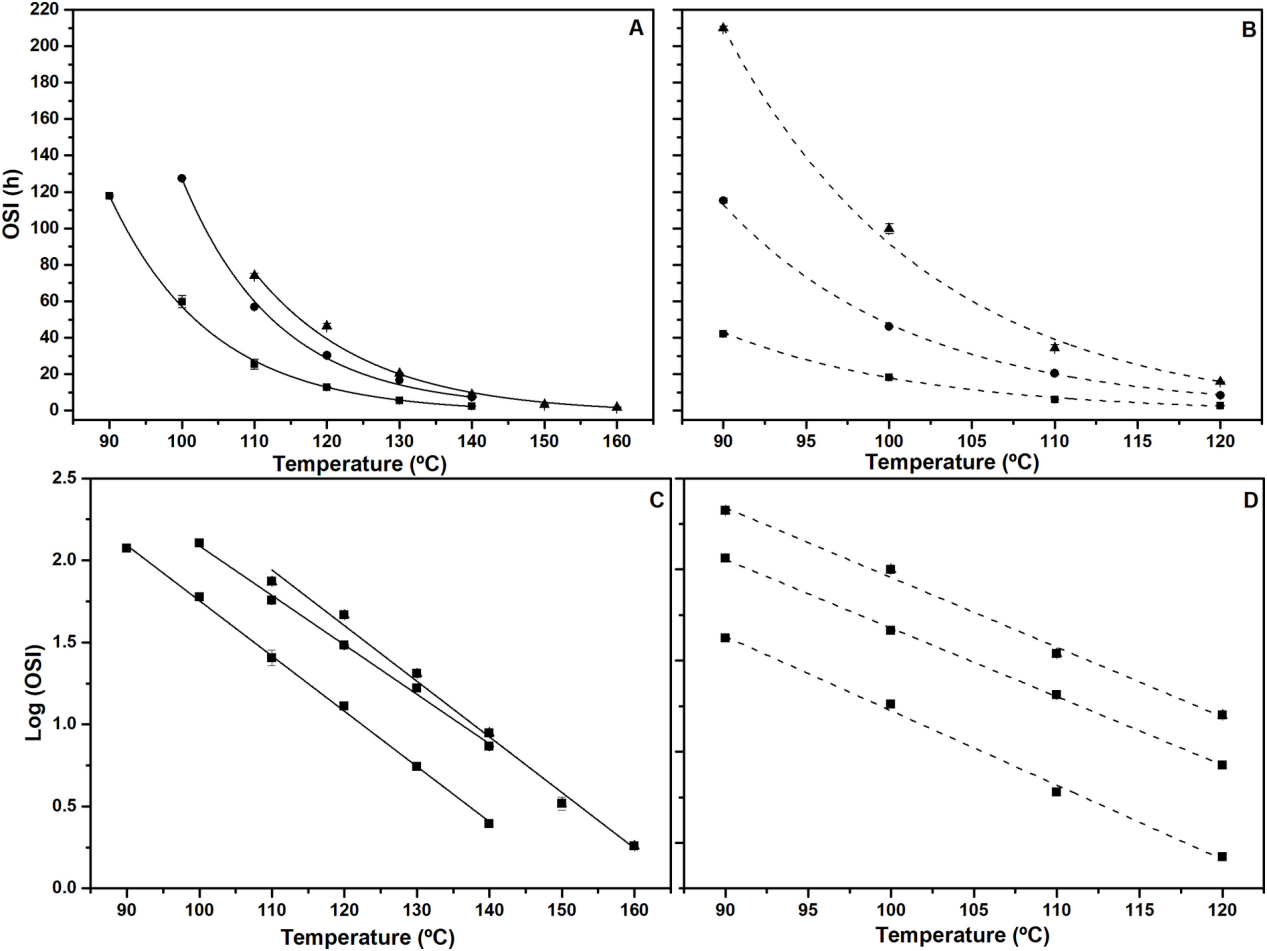
(Top) Oxidative stability index (OSI) measured at different temperature ranges by using accelerated conditions by the Rancimat (A) and Oxistest (B) methods for the extracted oils: cOO (squares), eOO (circles), and SCOO (triangles). (Bottom) Representation of Log (OSI) vs. temperature for cOO (squares), eOO (circles), and SCOO (triangles) by the Rancimat (C) and Oxitest (D) methods. Linear regression parameters A and B, from log OSI = At + B, are shown in Table 5.

Figure 4 shows the induction times or oxidative stability indexes (OSI) obtained at different temperatures for the extracted oils (cOO, eOO and SCOO) under accelerated conditions by the Rancimat (A) and Oxistest (B) methods. As mentioned above, the temperatures selected for each oil are based on its PC content, since the higher the PC content, the higher the temperature at which oxidative stability must be analyzed. It should be noted, however, that according to the literature, the closer the temperatures are to room temperature, the better the extrapolation at 20°C of the linear trend of log (OSI) vs. temperature (3).

As can be seen in Figure 4, OSI values decrease with increasing temperature. The high r^2^ values (0.991–0.999) obtained from the linear regression fits by using both Rancimat and Oxitest methods support to extrapolate the data at room temperature (20°C), and thus calculate the shelf life (OSI_20_) of the oils, which are shown in Table 5. Similar OSI_20_ results were obtained when using both accelerated methods without significant differences (*p* > 0.05).

**Table 5:** Linear regression (log OSI = At + B) parameters obtained for cOO, eOO, and SCOO using the Rancimat and Oxitest accelerated methods, as well as temperature coefficient (*t_coeff_*), *Q*_10_, and OSI_20_.

log OSI = A*t* + B					
	A = *t_coeff_* (^o^C^−1^)	B	R^2^	*Q*_10_	OSI_20_ (months)
Rancimat method					
cOO	−0.033 ± 0.000	5.124 ± 0.064	0.998 ± 0.001	2.17 ± 0.19	38.5 ± 1.7
eOO	−0.030 ± 0.001	5.103 ± 0.121	0.996 ± 0.001	2.05 ± 0.23	43.3 ± 0.2
SCOO	−0.034 ± 0.001	5.673 ± 0.209	0.991 ± 0.001	2.14 ± 0.43	138.6 ± 26.9
Oxitest method					
cOO	−0.041 ± 0.001	5.306 ± 0.183	0.995 ± 0.002	2.53 ± 0.43	42.5 ± 1.8
eOO	−0.038 ± 0.000	5.439 ± 0.059	0.999 ± 0.000	2.39 ± 0.12	67.0 ± 6.6
SCOO	−0.038 ± 0.001	5.782 ± 0.196	0.993 ± 0.000	2.39 ± 0.43	142.4 ± 1.9

These results confirm what it has been previously observed for VOO concerning the reduction of OSI values when using Oxitest, and the good correlation found at different temperatures (Figure 2B). Moreover, an alternative means to see the good correlation between the two methdos is by evaluating the Q_10_ values (see Table 5). No significant differences (p > 0.05) are found either between the two methodologies or between the different OOs, with values similar to those reported by Redondo-Cuevas et al. (42), ∼2.3 for OO.

In general, most oxidative stability studies reported for OO are carried out at a single temperature using an accelerated method, or under normal storage conditions at temperatures below 60°C (43). Given that these are EVOOs from the same variety and batch of olives, with similar quality indices, initial oxidative states (see Table 1), and with the same degree of instaurations (44), several factors, such as the chosen extraction processes and, consequently, the PCs content, will have a strong influence on the OSI values. In case of cOO, extracted by the conventional method, the OSIs obtained at 110°C (25.4 ± 2.8 h by Rancimat and 6.0 ± 0.3 h by Oxitest) (Figures 4A,B) are similar to those reported by Comandini et al. (27) for EVOO (21.8 and 6.7 h, respectively, using the same methodologies). Moreover, they agree with those obtained by Salvador et al. (28) at 100°C (∼ 60 h) by Rancimat for oils of the same variety and with similar amounts of PCs.

Kinetic analysis of the OSI measured for cOO in the temperature range 90–160°C by Rancimat and 90–120°C by Oxitest, represented as log (OSI) versus T (in °C), together with linear regression fits, are depicted in Figures 4C,D, respectively. Extrapolation to room temperature (20°C), provides the shelf-life (OSI20) for cOO of 38.5 and 42.5 months, when using Rancimat and Oxitest, respectively. These shelf-life values are substantially higher than the 28 months reported by Guillaume and Ravetti (45) for EVOO.

**Figure 5:**
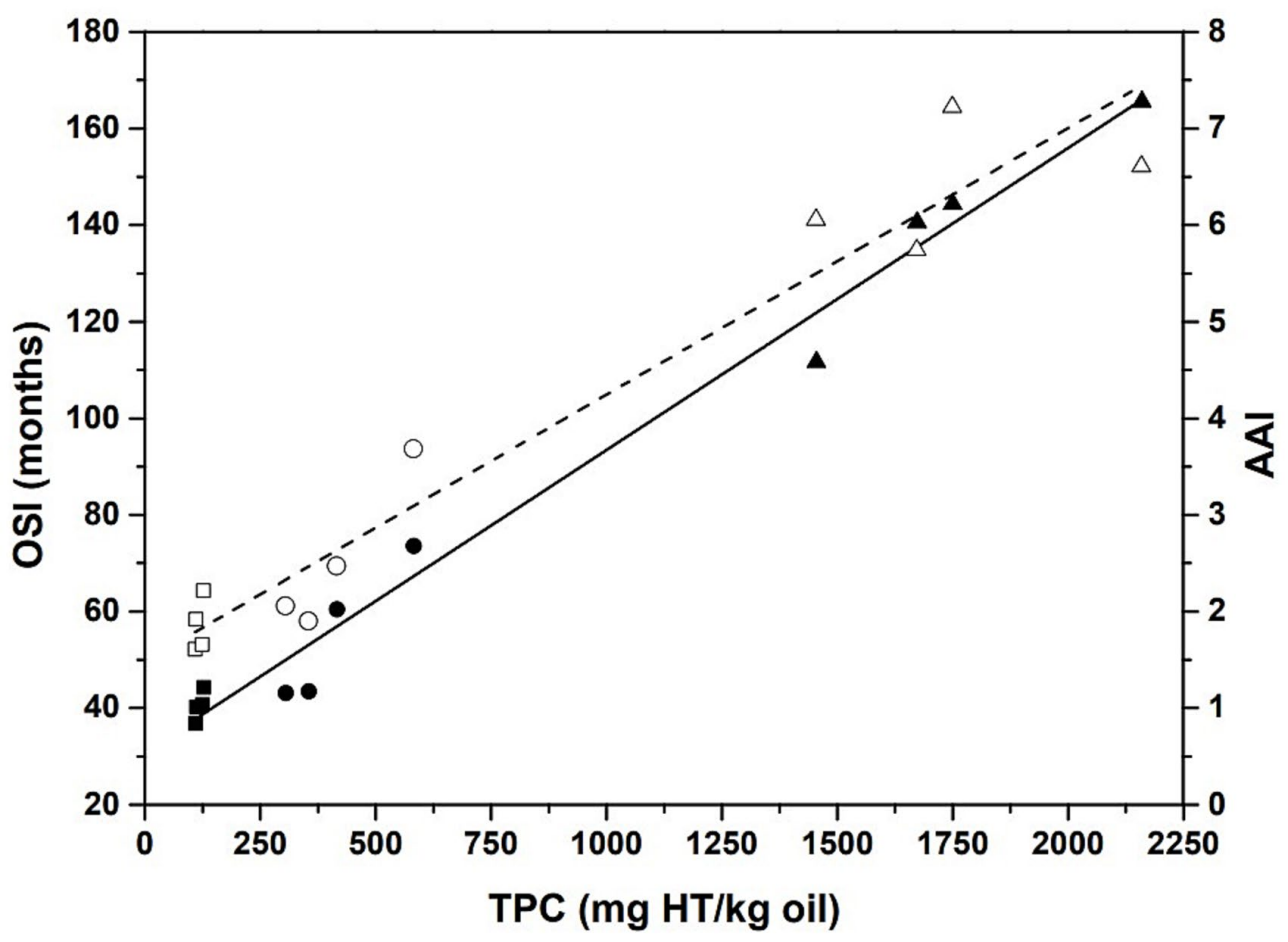
Liner regression of OSI20 values (filled black symbols) versus total phenolic compounds (TPC) (full line; r^2^ = 0.984) and antioxidant activity index (AAI) at 20°C (open symbols) vs. TPC (dashed line: r^2^ = 0.938). Symbols are squares for cOO, circles for eOO (circles), and triangles for SCOO, obtained by Rancimat and Oxitest methods.

The OSI_20_ values for eOO and SCOO were found to be 43.3 and 138.6 months for the Rancimat method, and 67 and 142 months for the Oxitest method, respectively. No shelf-life studies have been found in the literature for this type of oils. An oxidative stability study at 120°C by Rancimat for OO obtained by expeller press from dehydrated olives of the Moroccan Picholine variety was reported, and a value of OSI of 60.2 h was found (10). This is twice that obtained for eOO under the same conditions (Figure 4A). However, it must be considered that the content in PCs of this oil was approximately a factor of two higher. Del Carlo et al. (46) analyzed several EVOO samples by Rancimat at 100°C and the OSI values (5–24.4 h) were found to change as a function of the PC content (74–1,412 mg GAE/kg). The same correlation between OSI_20_ and the total phenolic content (TPC) has been found in the present work (see Figure 5), which would indicate a relationship between PCs content and OSI_20_ (months) that can be expressed for EVOO as,

(7) $${OSI}_{20}\;\left(\mathrm{months}\right)=0.0625\;TPC+30.905$$

with r^2^ = 0.984. This positive correlation was also obtained by Salvador et al. (28) and Nieto et al. (47) (r^2^ = 0.910) for oils of the same variety (Cornicabra) in different crop seasons, and by Artajo et al. (48) for refined OOs enriched with different amounts of PCs (*r*^2^ > 0.90).

The high content of PCs in the oils extracted by expeller press and SFE-CO_2_ makes the OSI_20_ of eOO and SCOO 1.3 and 3.5 times higher, respectively, than that for cOO. Once again, it is confirmed that the conventional extraction methodology and the presence of water have a negative effect on the oxidative stability of the oils (10, 49).

PCs contribute to the nutritional value and human health benefits of VOO, as well as its bitter flavor and antioxidant activity (50). The antioxidant activity of the different oils obtained was expressed as antioxidant activity indexes (AAIs), by comparing the OSIs of these oils with those of commercial OO. The oils under study contain natural PCs, such as secoiridoids derivatives (oleuropein), flavonoids (apigenin), and phenols (hydroxytyrosol and tyrosol). The antioxidant activity of these PCs has been related to their free radical scavenging properties, and specifically their ability to donate a hydrogen atom to the lipid radical formed during the propagation phase of lipid oxidation. Thus, they primarily work as chain breakers, donating a radical hydrogen atom to the alkyperoxyl radicals and subsequently forming a stable radical (48, 51).

The AAI at 20°C of the presently studied oils is above 1 (see Figure 5; cOO, 1.6–2.2; eOO, 1.9–3.7; SCOO, 5.7–7.2), and, therefore, all of them show protective effect. As can be seen in Figure 5, the higher the TPC content, the higher the AAI value, as well as the OSI20. This significant correlation between antioxidant capacity and TPC has also been reported by other authors (48, 52). In a study of the stability of VOO PCs during long-term storage at temperatures from 5 to 50°C, Krichene et al. (53) found that the degradation rate of PCs at 25°C is considerable, and it becomes faster as the storage temperature increases.

**Figure 6:**
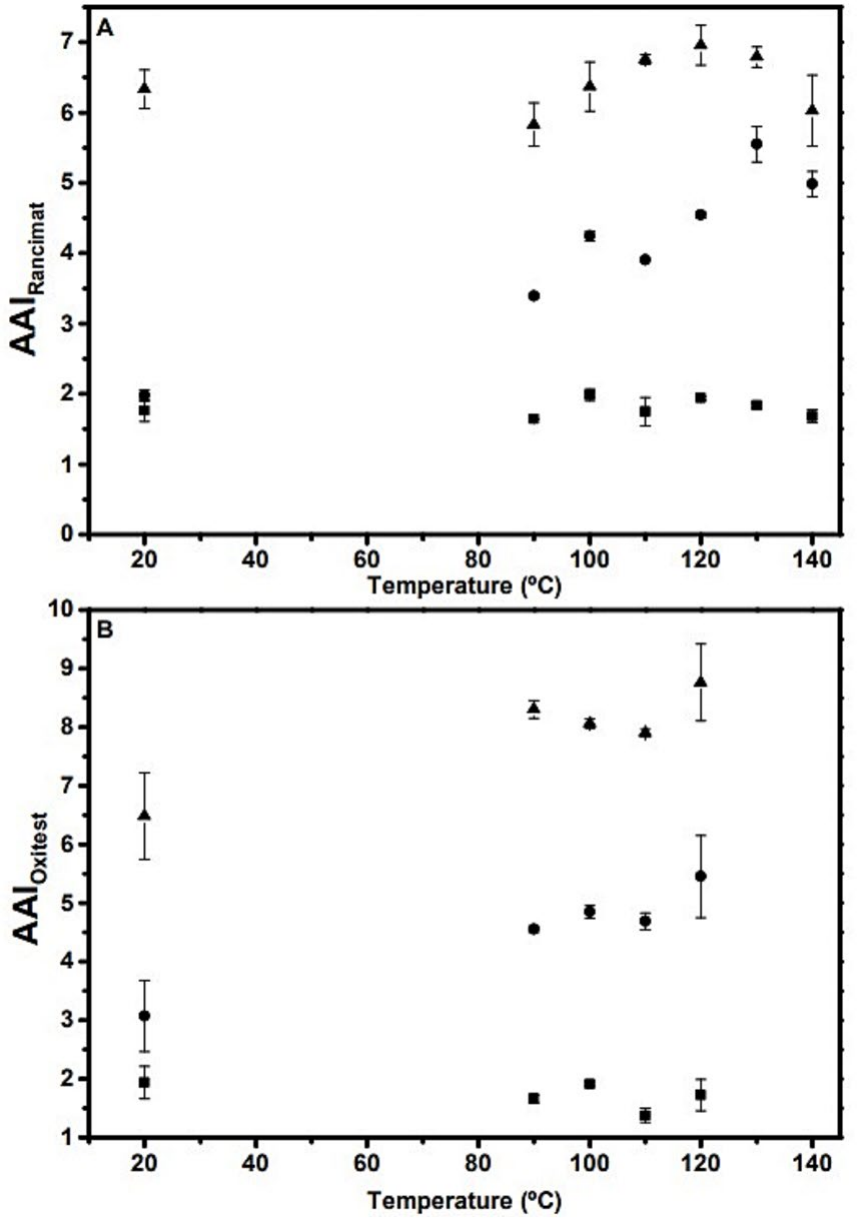
AAI values as a function of temperature in the range 90–140°C, for Rancimat (A), and 90–120°C for Oxitest (B) along with the extrapolation at 20°C. Squares: cOO. Circles: eOO. Triangles: SCOO.

Figure 6 show the temperature dependence of AAI for the different oils studied by the two accelerated methods, Rancimat and Oxitest. No significant change (*p* = 0.698) was observed in cOO by both Rancimat and Oxitest at different temperatures, which is in agreement with the results obtained by other authors for vegetable oils, whose tendency is to be stable with increasing temperature (23, 54). However, this behavior is not universally observed (55). Liu et al. (62) reported that AAI initially increases between 100–120°C, but then decreases at higher temperatures (130–150°C), which may indicate degradation of the PCs. This trend agrees with that obtained in the present study for eOO and SCOO by the Rancimat method (see Figure 6A), probably due to their higher antioxidant content. The antioxidant effect tends to peak at temperatures near decomposition (56). Since this study used 10°C intervals, the optimal antioxidant effect of eOO and SCOO could appear between 130–140°C and 120–130°C, with AAI values higher than 5.5 and 6.9, respectively.

In the case of Oxitest, the AAI trend of both oils is similar with higher values (6.1–9) from 90–120°C (see Figure 6B). This difference may be due to the different mechanism of action of the Rancimat and Oxitest tests. In Rancimat, the volatile compounds (carboxylic acids) are transferred to water, while in Oxitest, the consumption of oxygen is related to the formation of hydroperoxides (2, 4). Oxygen pressure reduces the partial volatility of antioxidant compounds and has no significant effect on AAI.

Finally, the differences in antioxidant capabilities of the cOO, eOO and SCOO oils is due to the extraction process and the content of hydrophilic PCs. The use of water in the extraction process of cOO makes that the content of PCs is very low. However, when using expeller press, a first part is extracted in the eOO and a second part is dragged in the extraction by CO_2_ to obtain SCOO. The antioxidant power of the hydrophilic PCs is high since they cover and orient in the air-liquid interface and thus they protect the oil against oxidation. However, the lithophilic compounds remain in solution in the oil (4, 5). Therefore, these oils, and especially SCOO, have an added value to be used as antioxidants for other lipids and foods.

### 3.6 Arrhenius kinetics and activation thermodynamic magnitudes of the extracted oils

**Figure 7:**
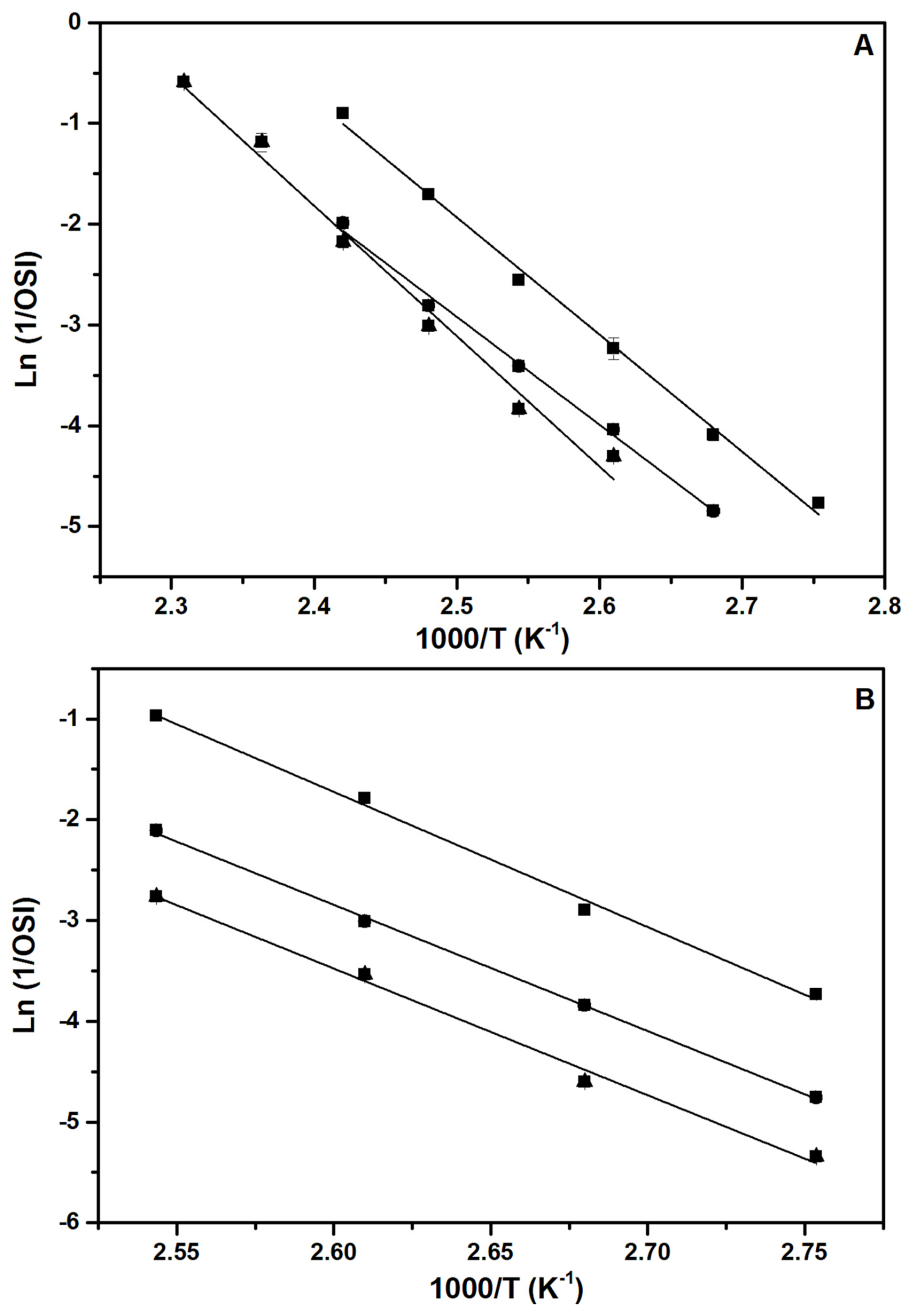
Representation of Ln k vs. 1/T, where k = 1/OSI, for (A) cOO (squares), eOO (circles), and SCOO (triangles) obtained by the Rancimat test, and for (B) the same oils but using the Oxitest method. The straight lines in each panel correspond to the linear regression of the data. The fitting parameters (slope and intercept) are listed in Table 6. Error bars are smaller than the size of the symbols in each case.

Temperature is one of the most important factors affecting lipid oxidation. Oxidative stability is defined in terms of a reaction rate constant (k) which defines the degradation of the lipid matrix by the formation of oxidation products (43, 57). The quantitative relationship between k and temperature T, is expressed in terms of the Arrhenius law (see Equation 3). Figure 7 shows the representation of the linear relationship between Lnk, which in the present case corresponds to the reciprocal OSI (h^−1^), i. e., Ln k = Ln(1/OSI), and 1/T (in K^−1^), according to Equation 3, for all the oils and accelerated methods. The fits provide the activation energy (Ea) and the pre-exponential factor (A) for each case, which are shown in Table 6 together with the fitting parameters (slope and intercept).

**Table 6:** Arrhenius kinetic data and thermodynamic activation parameters for each oil (cOO, eOO and SCOO) and accelerated method (Rancimat and Oxitesr): Pre-exponential factor (*A*), activation energy (*E_a_*), activation enthalpy (ΔH^‡^), activation entropy (ΔS^‡^) and activation Gibb’s free energy (Δ*G*^‡^).

Oil samples	Ln *k* = Ln(1/OSI) = a + b/T							
	a (intercept)	b (slope)	R^2^	*A* (h^−1^)	*E_a_* (kJ·mol^−1^)	Δ*H^‡^* (kJ·mol^−1^)	Δ*S^‡^* (J·mol^−1^·K^−1^)	ΔG*^‡^* (kJ·mol^−1^)
Rancimat method								
cOO	27.1 ± 0.96	−11.62 ± 0.37	0.995	9.6.1·10^11^	96.6 ± 3.1	93.4 ± 3.1	−29.9 ± 7.9	104.9 ± 0.03
eOO	23.8 ± 1.01	−10.69 ± 0.39	0.995	3.47·10^10^	92.9 ± 3.3	85.7 ± 3.2	−57.4 ± 8.4	107.7 ± 0.1
SCOO	29.2 ± 1.8	−12.93 ± 0.73	0.984	1.54·10^13^	107.5 ± 6.1	104.1 ± 6.1	−12.8 ± 14.9	109.1 ± 0.36
Oxitest method								
cOO	33.1 ± 1.58	−13.41 ± 0.59	0.995	5.11·10^13^	106.5 ± 5.0	108.4 ± 4.9	−20.4 ± 13.1	100.5 ± 0.1
eOO	29.7 ± 0.75	−12.52 ± 0.24	0.999	9.8·10^12^	104.1 ± 2.0	101 ± 1.9	−8.03 ± 5.16	104.1 ± 0.03
SCOO	29.2 ± 1.79	−12.57 ± 0.67	0.991	1.48·10^13^	104.5 ± 5.6	101.3 ± 5.6	−12.4 ± 14.8	106.1 ± 0.08

As can be seen in Figure 7 and Table 6, high correlation factors (R^2^) are obtained in all the cases, i. e. different oils studied, and acceleration method used. The activation energies Ea obtained for cOO, eOO and SCOO are in the range 96.6–107.5 kJ mol^−1^ and the pre-exponencial factors A range from 3.47·10^10^ to 5.11·10^13^ h^−1^, without showing significant differences between the two accelerated methods used (Rancimat and Oxitest). These Ea values are comparable to those reported for OO (97.7–102 kJ mol^−1^) and those for other vegetable oils at higher temperatures (100–140°C) (57, 58). Soybean and sunflower oils have Ea values of 92.4 and 90.7 kJ·mol^−1^, respectively, at temperatures between 100 and 130°C (18), while avocado and coconut oils have Ea of 99.6 and 86.9 kJ·mol^−1^, respectively, at temperatures between 110 and 140°C (59). Large values of Ea are indicative of reactions which are more temperature-dependent, i. e., reactions which require high temperatures and, thus, larger collision frequencies for reactive change to occur (60). The large values of Ea found for the oils under study are related with their high antioxidants content. This is so because the antioxidants present in the oils act as peroxyl radical scavengers, delaying lipid oxidation and the formation and decomposition of hydroperoxides (57).

Regarding activation thermodynamic magnitudes, enthalpy (ΔH^‡^), entropy (ΔS^‡^) and Gibbs’ free energy (ΔG^‡^), were determined by using Equations 4, 5 and are listed in Table 6 for all the oils under study and accelerated methods used. As can be seen, all investigated oils have positive ΔH^‡^ values, confirming the endothermic character of the lipid oxidation mechanism (18, 58). The ΔH^‡^ values range from 85.7 kJ mol^−1^ for oil eOO to 108.5 kJ mol^−1^ for cOO, and the ΔS^‡^ values range from −29.9 J mol^−1^ K^−1^ for cOO to −8.03 J mol^−1^ K^−1^ for eOO. Farhoosh and Hoseini-Yazdi (18) reported ΔH^‡^ and ΔS^‡^ values of 83.64 kJ mol^−1^ and -116.66 J mol^−1^ K^−1^, respectively, for OO. Gharby et al. (31) obtained values similar to those obtained in the present work for OO, with ΔH^‡^ ranging from 86.21 to 98.44 kJ mol^−1^ and, ΔS^‡^ ranging from −50.5 to −14.5 J mol^−1^ K^−1^.

The activation Gibbs’ free energy (ΔG^‡^) was obtained at 110°C using Equation 5 (see Table 6) and ranges from 100.5 to 109.1 kJ mol^−1^ for the extracted oils, which represents the balance of the positive ΔH^‡^ and negative ΔS^‡^ values. These positive values for ΔG^‡^ confirm the non-spontaneous, endergonic and endothermic lipidic oxidation process, demonstrating that the oils extracted by expeller and SFE-CO_2_ do not behave differently from those extracted in the conventional way.

## 4 Conclusion

The present work provides significant information about the Rancimat and Oxitest accelerated methods to study the oxidative stability of olive oils obtained by different extraction methods, i.e., conventional 2-phase extraction (cOO), expeller press extraction (eOO), and supercritical CO_2_ extraction (SCOO). The studied oils showed quality indexes within the legal limits established for VOO and EVOO, as well as similar fatty acid profiles, suggesting that the extraction method has no impact on these parameters. Although all the oils studied showed good quality and stability, the extraction method clearly influences the phenolic content of the oil, affecting thus to its oxidative stability. Estimated shelf-life (OSI_20_) values indicate that the oil obtained by supercritical CO_2_ extraction shows a higher phenolic content and longer shelf life, which make it potentially superior for long-term storage and use. In addition, it should be noted the enormous potential of this oil as a biological and technological antioxidant.

Finally, it should be mentioned that Oxitest provides OSI values lower than those attained by Rancimat, since Oxitest measures the early stages of lipid oxidation. However, the results obtained by the two accelerated methods correlate very well. As an additional advantage, Oxitest requires less sample quantity. Considering all above, we conclude that Oxitest can be a good and faster alternative to Rancimat for the evaluation of oil oxidation stability.

## Data Availability

The original contributions presented in the study are included in the article/supplementary material, further inquiries can be directed to the corresponding author.
